# Effectiveness of Bimekizumab in Multi-Failure Psoriatic Patients: A Retrospective, Real-World Multicenter Study

**DOI:** 10.3390/jpm16010027

**Published:** 2026-01-05

**Authors:** Francesca Satolli, Giulia Rech, Silvia Gerosa, Laura Bigi, Andrea Conti, Vito Di Lernia, Claudia Lasagni, Rosita Longo, Michela Tabanelli, Federico Bardazzi

**Affiliations:** 1 Dermatology Unit, Department of Medicine and Surgery, University of Parma, 43121 Parma, Italy; 2 Division of Dermatology, Psoriasis Outpatient Service, APSS (Azienda Provinciale per i Servizi Sanitari), 38122 Trento, Italy; 3 Dermatology Unit, Department of Surgical, Medical, Dental & Morphological Sciences with Interest Transplant, Oncological & Regenerative Medicine, University of Modena and Reggio Emilia, 41121 Modena, Italy; 4 Dermatologic Unit, Department of Surgery, Infermi Hospital of Rimini, auSL Romagna, 47923 Rimini, Italy; 5 Dermatology Unit, Arcispedale Santa Maria Nuova, Azienda USL-IRCCS di Reggio Emilia, 42123 Reggio Emilia, Italy; 6 Dermatology Unit and Regional Skin Bank, 47521 Cesena, Italy; 7 Dermatologic Unit, Department of Surgery, Santa Maria Delle Croci Hospital of Ravenna, AUSL Romagna, 48121 Ravenna, Italy; 8 Division of Dermatology, Department of Experimental, Diagnostic and Specialty Medicine, S. Orsola-Malpighi Hospital, University of Bologna, 40138 Bologna, Italy

**Keywords:** bimekizumab, multi-failure patients, plaque psoriasis, real-life study

## Abstract

**Background/Objectives:** Patients with moderate-to-severe psoriasis who experience inadequate response or loss of efficacy to multiple biologic agents (“multi-failure patients”) represent a particularly challenging subgroup in clinical practice. Evidence regarding the efficacy of bimekizumab in this setting is still limited. This multicentre, real-life study aimed to evaluate the effectiveness, safety, and treatment persistence of bimekizumab in patients with moderate-to-severe psoriasis who had failed at least two previous biologic therapies. **Methods:** This multicentre, retrospective, real-life study across Italian referral centers retrospectively collected clinical data from 33 adult patients with plaque psoriasis treated with bimekizumab across Italian referral centers. Efficacy was assessed through changes in Psoriasis Area and Severity Index (PASI) and Dermatology Life Quality Index (DLQI) scores at weeks 4 and 16. Logistic regression was performed to identify predictors of treatment response, and Kaplan–Meier analysis evaluated drug survival up to 12 months. **Results:** The mean baseline PASI was 14.5 ± 7.1, decreasing to 1.5 ± 4.0 at week 16 (*p* < 0.001). PASI90 and PASI100 responses were achieved by 57.6% and 42.4% of patients at this timepoint, respectively, while mean DLQI improved by 84.2%. In this small cohort, no significant differences in efficacy were observed according to the number or class of prior biologic failures. Genital psoriasis was associated with a lower likelihood of achieving PASI100. Adverse events were generally mild to moderate in severity and manageable in routine clinical practice. No discontinuations occurred due to lack of efficacy; all withdrawals were related to mild adverse events or personal reasons. Twelve-month drug survival reached 85.4% (95% CI 63.8–100). **Conclusions:** Bimekizumab demonstrated rapid, marked, and sustained clinical improvements with a favorable safety profile in multi-failure psoriasis patients. These findings support its role as an effective and well-tolerated therapeutic option for individuals with highly refractory disease in real-life practice.

## 1. Introduction

The introduction of biological treatments has completely changed the management of moderate-to-severe psoriasis, achieving higher levels of efficacy and safety as compared with conventional systemic agents [1]. Recently, 12 biologics drugs have been approved targeting tumor necrosis factor (TNF) alpha, interleukin (IL) 12/23, IL-17, and IL-23 [2]. These targeted agents have transformed treatment goals from partial improvement to complete or near-complete skin clearance, deeply impacting patients’ quality of life and long-term disease control [2]. Moreover, this wide therapeutic armamentarium now available enables a more personalized management approach, allowing clinicians to tailor drug selection according to individual patient profiles, comorbid conditions, and clinical history [3]. Nevertheless, there is still a proportion of patients who remains difficult-to-treat, as they experience an inadequate response or progressive loss of efficacy to multiple biologics [4]. These subjects, commonly referred to as “multi-failure patients”, have become a clinical challenge in daily clinical practice. Nowadays, there are no shared guidelines or consensus which may suggest the most appropriate therapeutic strategies, mainly the parameters for switching among biological drugs of the same or different classes [5,6,7]. Furthermore, the definition of ‘multi-failure patients’ remains inconsistent across studies, as the number of biologic therapies that must have failed before classifying a patient as such varies widely among reports [5,6,7].

Bimekizumab, a humanized monoclonal IgG1 antibody, is the most recently approved biologic for the treatment of moderate-to-severe plaque psoriasis [8]. It is characterized by a unique mechanism of action, selectively targeting both IL17-A and IL-17F. This dual inhibition differentiates bimekizumab from other IL-17 pathway inhibitors, such as secukinumab and ixekizumab, which target IL-17A alone, and brodalumab, which binds to the IL-17 receptor A (IL-17RA) [8]. By simultaneously inhibiting IL-17A and IL-17F, bimekizumab targets two synergistic cytokines involved in residual inflammatory activity, a mechanism that may be particularly relevant in patients with refractory disease.

Since its approval, numerous real-world studies have consistently confirmed the high effectiveness and favorable safety profile of bimekizumab across heterogeneous patient populations, including those with psoriatic arthritis (PsA), metabolic or renal comorbidities, obesity, elderly individuals, and patients with involvement of difficult-to-treat areas such as the scalp, nails, and genital region, as well as in those previously exposed to other biologics [9]. These real-life data are mandatory because they reflect the heterogeneity of everyday clinical practice, often including older patients previously excluded from randomized trials [10].

Despite the growing of real-world evidence, studies specifically investigating the use of bimekizumab in patients with multiple prior biologic failures are still absent [9]. Available reports often include mixed populations, and subgroup analyses by treatment history remain limited [9].

Assessing the effectiveness of bimekizumab in this difficult-to-treat population is clinically relevant since it may guide therapeutic sequencing and improve treatment algorithms.

In this context, the aim of this study was to evaluate the effectiveness and safety of bimekizumab in a real-life cohort of patients with moderate-to-severe plaque psoriasis who had experienced multiple biologic failures. Specifically, the main aim was to evaluate the efficacy and safety of bimekizumab in patients with moderate-to-severe plaque psoriasis who had previously failed at least two biologic therapies following 16 weeks of treatment. Particular attention was paid to the subgroup of patients with multiple biologic failures (≥4), a population for which clinical evidence remains limited.

Secondary outcomes included the assessment of the impact of bimekizumab on health-related quality of life, the evaluation of treatment response according to previous biologic exposure, the identification of predictors of therapeutic response, and the characterization of safety outcomes during treatment. In addition, a post hoc exploratory analysis was performed to evaluate drug survival and long-term treatment persistence beyond the 16-week primary endpoint.

## 2. Material and Methods

A multicentre, real-life, retrospective, observational study was conducted including adult patients with moderate-to-severe plaque psoriasis who initiated treatment with bimekizumab in routine clinical practice. Data were collected from 8 Italian dermatology referral centres. Inclusion criteria were: patients aged ≥18 years, dermatologist-confirmed diagnosis of plaque psoriasis, treatment with bimekizumab, availability of at 16 weeks of follow-up at the time of data collection, and failure of at least two prior biologic therapies due to primary inefficacy, secondary loss of response, or adverse events. Although no universally accepted definition of “multi-failure” exists, this threshold was selected to reflect real-world clinical practice. All patients received bimekizumab according to the standard approved dosing regimen; no dose intensification, delayed injections, or off-label dosing strategies were applied.

For each patient, baseline demographic and clinical characteristics were recorded, including age, sex, body mass index (BMI), and year of psoriasis onset. Clinical data included disease duration, presence of PsA, and involvement of difficult-to-treat areas such as scalp, nails, and genital regions. The presence of comorbid conditions was documented and categorized as metabolic (including diabetes mellitus, obesity, dyslipidaemia), cardiovascular (hypertension, heart disease), hepatic (HBV/HCV-related liver disease), endocrine (thyroid disorders), oncologic, autoimmune/inflammatory, or psychiatric. Comorbidities were identified through retrospective medical chart review and were based on previously documented specialist diagnoses. Previous systemic and biologic treatments were reported in detail, including both the drug class and the duration of each therapy in months.

Efficacy was evaluated through changes in Psoriasis Area and Severity Index (PASI) and Dermatology Life Quality Index (DLQI) scores collected at baseline, week 4, and week 16. The primary efficacy endpoint was the improvement in the PASI score and DLQI score at week 4 and week 16. Secondary outcomes included the proportion of patients achieving PASI90 and complete skin clearance (PASI100) at week 4 and week 16, the mean percentage reduction in PASI and DLQI from baseline, and the assessment of treatment discontinuation rates and their underlying causes. Lastly, a sub analysis to evaluate drug survival up to 12 months of therapy was performed.

### Statistical Analysis

Continuous variables were expressed as mean ± standard deviation, while categorical variables were presented as absolute frequencies and percentages. Statistical analyses included both descriptive and inferential approaches. Differences in mean PASI and DLQI scores between baseline and follow-up visits (weeks 4 and 16) were analysed using paired *t*-tests to assess the significance of clinical improvement over time. When relevant, unpaired *t*-tests and Chi-square tests were employed to compare continuous and categorical variables between subgroups. Notably, analyses of PASI and DLQI changes over time were performed using complete-case data; no imputation methods were applied for missing values.

To identify potential predictors of therapeutic response, a multivariate logistic regression analysis was conducted with PASI100 achievement at week 16 as the dependent variable. Independent variables included age, sex, BMI, presence of PsA, involvement of difficult-to-treat areas (nail, scalp, genital), and the number of previously failed biologic therapies. A separate logistic regression model was also performed considering PASI90 as the outcome variable. Odds ratios (ORs) with corresponding 95% confidence intervals (CIs) and *p*-values were calculated and reported. All prespecified covariates were entered simultaneously into the multivariate logistic regression models; no variable selection or stepwise procedures were applied.

Drug survival was assessed using the Kaplan–Meier method. Discontinuations occurring within the first 12 months of therapy were considered events, whereas patients who continued treatment beyond 12 months were censored. The probability of remaining on bimekizumab treatment at 12 months was expressed as a survival percentage with 95% CIs, estimated according to Greenwood’s formula. Given the limited sample size, all regression analyses were considered exploratory and hypothesis-generating. The results should therefore be interpreted with caution.

This study was conducted in accordance with the principles of the Declaration of Helsinki and with local regulatory requirements. Given its retrospective design, observational nature, and the use of anonymized data, the study protocol did not require formal approval by an institutional ethics committee, and patient informed consent was waived.

All statistical analyses were performed using GraphPad Prism (version 8.0, GraphPad Software, La Jolla, CA, USA) and Python (version 3.11) statistical libraries. A two-tailed *p*-value < 0.05 was considered statistically significant.

## 3. Results

A total of 33 patients with moderate-to-severe plaque psoriasis who initiated bimekizumab therapy following the failure of at least 2 biologics were included in the present analysis. The mean age at treatment initiation was 56.2 ± 12.8 years, and 18 (54.5%) patients were male. The mean BMI was 29.8 ± 5.0 kg/m^2^, reflecting an overweight population. The mean duration of psoriasis was 29.1 ± 13.0 years, indicating a long-standing and treatment-refractory disease course.

About half of the patients (15/33, 51.5%) had concomitant psoriatic arthritis (PsA). Involvement of difficult-to-treat areas was common: scalp psoriasis was reported in 23 (69.7%), nail psoriasis in 11 (33.3%), and genital psoriasis in 13 (39.4%) patients, respectively. These findings reflect a cohort with a complex disease phenotype and multiple refractory sites.

Comorbidities were highly prevalent, with metabolic disorders (including diabetes mellitus, obesity, or dysmetabolic syndrome) as the most common (*n* = 13, 39.4%), followed by cardiovascular disease in (*n* = 11, 33.3%), oncologic disorders (*n* = 6, 18.2%), hepatic disease (HBV/HCV-related) (*n* = 4, 12.1%), autoimmune/inflammatory disorders (*n* = 4, 12.1%), endocrine disorders (*n* = 3, 9.1%), and psychiatric disorders (*n* = 2, 6.1%). These data confirm the high systemic burden of comorbidities typically observed in real-world psoriasis populations.

Among the 33 patients included, one (3.0%) patient had failed exactly two biologic therapies and one (3.0%) had failed three, whereas the majority (29/33; 87.9%) had failed four or more biologics. Specifically, 14 (42.4%) patients had failed four biologics, 15 (45.5%) had failed five, and 2 (6.1%) had failed six biologics. The most frequently used previous biologics were adalimumab (*n* = 28, 84.8%), secukinumab (*n* = 19, 57.6%), ixekizumab (*n* = 16, 48.5%), ustekinumab (*n* = 13, 39.4%), guselkumab (*n* = 13, 39.4%), infliximab (*n* = 11, 33.3%), and etanercept (*n* = 10, 30.3%). Of note, despite inclusion criteria required failure of at least two biologic therapies, the vast majority of patients (29/33; 87.9%) had failed four or more biologic agents, thus representing a highly treatment-refractory population. Exclusion of the two patients with fewer than four prior biologic failures did not result in meaningful differences in efficacy or safety outcomes.

Smaller proportions had been exposed to tildrakizumab (*n* = 8, 24.2%), certolizumab (*n* = 4, 12.1%), and golimumab (*n* = 1, 3.0%). The mean duration of treatment with previous biologics ranged from 7.1 ± 3.9 months for brodalumab to 27.6 ± 33.3 months for ustekinumab, reflecting heterogeneous persistence and multiple sequential therapeutic failures prior to switching to bimekizumab. Although the mean duration of several prior biologic therapies exceeded one year, treatment discontinuation was mainly driven by secondary loss of efficacy or the occurrence of adverse events, rather than by sustained disease control. Baseline demographic and clinical characteristics, comorbidities, previous biologic exposure, and clinical outcomes of enrolled patients were summarized in Table 1.

### 3.1. Efficacy Outcomes

At baseline, the mean PASI score was 14.5 ± 7.1, decreasing to 2.5 ± 2.9 at week 4 and 1.5 ± 4.0 at week 16 (*p* < 0.001 for both comparisons vs. baseline). Similarly, DLQI improved from 15.7 ± 5.0 at baseline to 2.3 ± 3.0 at week 4 and 2.0 ± 3.6 at week 16 (*p* < 0.001 for both). This corresponds to a mean PASI reduction of 79.7% at week 4 and 90.4% at week 16, in line with a DLQI improvement of 86.9% and 84.2%, respectively.

In detail, 14 (42.4%) patients achieved PASI90, and 7 (21.2%) achieved PASI100 after only one month of treatment. At week 16, these proportions increased to 19 (57.6%) and 14 (42.4%) patients, respectively. The improvement in PASI and DLQI scores was statistically significant at each follow-up, suggesting both the rapid and sustained clinical benefit of bimekizumab.

When stratified by treatment history, response rates remained consistently high across subgroups. Among patients who had failed four biologics (*n* = 8), 6 (75.0%) achieved PASI90 and 4 (50.0%) achieved PASI100 at week 16. Among those who had failed five or more biologics (*n* = 13), 11 (84.6%) and 9 (69.2%) reached PASI90 and PASI100, respectively. Of note, no statistically significant differences between groups. Descriptively, short-term efficacy outcomes at week 16 in patients with two or three prior biologic failures did not differ meaningfully from those observed in patients with ≥4 biologic failures. These findings indicate that bimekizumab maintained high efficacy also in heavily pretreated and biologic-experienced patients.

In the multivariate logistic regression model, genital involvement was associated with a lower likelihood of achieving complete skin clearance (PASI100) at week 16 (OR = 0.01; 95% CI 0.00–0.97; *p* = 0.048); however, this association should be interpreted cautiously due to model instability related to the small sample size. No other baseline variable, including age, sex, BMI, PsA, nail or scalp involvement, or number of previous biologics, was significantly associated with the likelihood of PASI100.

In a secondary model exploring predictors of PASI90, no variable reached statistical significance.

### 3.2. Safety and Drug Survival

The most common adverse event was oral candidiasis, occurring in 5 (15.2%) patients. Other reported events included infectious complications such as erysipelas and urinary tract infections (*n* = 1, 3.0%), psodermatitis (*n* = 1, 3.0%), lichen ruber planus (*n* = 1, 3.0%), generalized eczema (*n* = 1, 3.0%), and worsening of PsA (*n* = 1, 3.0%). All adverse events were mild to moderate in severity, and no serious or unexpected safety concerns emerged during follow-up. During the 16-week treatment period, a total of 4 (12.1%) patients discontinued bimekizumab. Three withdrawals occurred early (at week 4), two due to adverse events and one for personal reasons, while one patient discontinued at week 16 because of generalized eczema. None of the early discontinuations were related to lack of efficacy.

Although the primary analysis focused on outcomes up to week 16, an exploratory post hoc assessment was conducted to evaluate drug persistence beyond this timepoint. This additional analysis confirmed that the majority of patients maintained bimekizumab treatment over time. In detail, the mean duration of follow-up was 12.7 ± 6.5 months, with a maximum follow-up time of 28 months. At the 12-month landmark, Kaplan–Meier analysis showed a drug survival of 85.4% (95% CI 63.8–100). Most patients maintained treatment beyond one year, with only a few early discontinuations contributing to drug withdrawal. In detail, a total of six patients (18.2%) discontinued bimekizumab. Three of them interrupted treatment due to adverse events [oral candidiasis (*n* = 2, 6.1%); generalized eczema (*n* = 1, 3.0%)], two due to personal reasons, and one for an unspecified cause; notably, no patient discontinued because of loss of efficacy. Of note, cases of oral candidiasis were diagnosed based on clinical assessment during routine dermatological follow-up; microbiological confirmation was not routinely required in clinical practice. Among patients who developed oral candidiasis, some were able to continue bimekizumab after appropriate antifungal treatment, whereas others discontinued therapy, as detailed above. It should be highlighted that at the 12-month landmark, 19 (57.6%) patients had reached at least 12 months of follow-up, while the remaining patients were censored at earlier timepoints due to shorter observation periods.

## 4. Discussion

Patients with psoriasis who experience inadequate response or loss of efficacy to multiple biologic agents, often called “multi-failure patients”, represent a particularly challenging and understudied population in clinical practice [5,6,7].

In our real-world study including 33 patients with moderate-to-severe psoriasis who had failed at least two biologics, most of whom (29/33; 87.9%) had failed four or more agents, bimekizumab achieved rapid and marked efficacy, with PASI90 and PASI100 responses achieved by 57.6% and 42.4% of patients at week 16, respectively. DLQI significantly improved at each timepoint as well. Notably, our population had unfavourable baseline profile, characterized by a mean disease duration of nearly 30 years, a high prevalence of PsA (51.5%), and frequent involvement of challenging sites such as the scalp (69.7%), nails (33.3%), and genital area (39.4%). In this small real-world cohort, we did not observe significant differences in efficacy according to prior biologic exposure. Importantly, no discontinuations were attributed to loss of efficacy, as all treatment interruptions occurred due to mild adverse events or personal reasons, further supporting the sustained effectiveness of bimekizumab in this difficult-to-treat population.

Recently, an Italian multicenter retrospective study including 1154 patients with psoriasis (97 classified as multi-failure) tried to systematic characterize this difficult-to-treat population [11]. The authors defined multi-failure arbitrarily as patients who had failed at least four biologic therapies, acknowledging that no standardized definition currently exists in the literature [11]. Compared with the overall cohort, multi-failure patients exhibited distinct clinical features, including a significantly earlier age at psoriasis onset (29.7 ± 14.1 vs. 35.1 ± 14.4 years; *p* = 0.001), higher prevalence of PsA (45.4% vs. 26.9%; *p* = 0.001), diabetes (30.9% vs. 10.9%; *p* < 0.001), and cardiovascular comorbidities (54.6% vs. 39.8%; *p* = 0.008) [11]. Notably this study did not include patients treated with bimekizumab [11]. Moreover, the authors observed a reduction in treatment response in multi-failure subjects, despite initial improvements at week 16 [11]. In our cohort, the exploratory post hoc assessment confirmed the effectiveness of bimekizumab treatment over time, showing a drug survival of 85.4% (95% CI 63.8–100) at 52 weeks.

In the literature, there are several real-life experiences confirming the effectiveness of bimekizumab, without significant differences between bio-naïve or bio-experienced patients [12,13,14,15,16,17]. Our study, specifically focusing on multi-failure patients, reinforces these data. Notably, efficacy was preserved regardless of both the number and the class of previously failed biologics, reinforcing the concept that dual IL-17A and IL-17F inhibition may overcome partial resistance mechanisms observed with prior anti-IL-17 or anti-IL-23 therapies.

Treatment persistence is another critical parameter in evaluating biologic performance in real-world contexts. In our exploratory survival analysis, the 12-month drug survival rate was 85.4%, with most patients maintaining therapy well beyond one year. This result is particularly relevant given that our population consisted predominantly of patients with multiple previous treatment failures, who typically exhibit lower persistence rates. Comparable long-term data reported that bimekizumab demonstrated high retention rates and durable PASI90/100 responses through 52 weeks [16,17]. These findings indicate that bimekizumab maintains efficacy and tolerability over time, even in highly pretreated patients.

It should be noted that our cohort also included a high proportion of patients with PsA (51.5%), metabolic and cardiovascular comorbidities, and prior oncologic or hepatic disease, reflecting the systemic burden typical of real-life psoriasis populations. Despite these complexities, bimekizumab was well tolerated, with no serious or unexpected adverse events. Moreover, oral candidiasis was the most frequent adverse event (15.2%), in line with the incidence reported in both clinical trials and real-world studies (3–21%) [18].

Another relevant finding from our study was the identification of genital involvement as an independent negative predictor of achieving complete skin clearance (PASI100) at week 16 (OR = 0.01; 95% CI 0.00–0.97; *p* = 0.048). Differently, IL PSO data on genital psoriasis, showed that bimekizumab led to marked improvements in Static Physician Global Assessment of Genitalia score (100% clear or almost clear) at week 16, without significant differences regardless of prior exposure to biologics [19]. Compared with the recent real-world study by Esposito et al. that identified lower baseline PASI, absence of nail involvement, and fewer prior biologic failures as predictors of early complete clearance, our analysis found no significant baseline factors associated with PASI90 or PASI100 at week 16, except for genital psoriasis, which emerged as an independent negative predictor of achieving complete skin clearance [20].

From a mechanistic perspective, our results further support the biological rationale for dual IL-17A and IL-17F blockade in refractory psoriasis [21]. Both cytokines are abundantly expressed in lesional skin and act synergistically to amplify the proinflammatory cascade. While IL-17A inhibition alone provides substantial efficacy, IL-17F contributes independently to residual disease activity, and its suppression may explain the deeper and more consistent skin clearance achieved with bimekizumab [21]. Moreover, IL-17F blockade might reduce the recurrence of low-grade inflammation that underlies secondary loss of response in patients switched from other IL-17A inhibitors [21].

## 5. Strengths and Limitations

The main strengths of this study lie in its real-world, multicentre design and in the inclusion of a cohort of highly treatment-refractory patients who had failed multiple biologic lines before initiating bimekizumab. This allowed for the evaluation of the drug’s effectiveness and safety in a population that is typically underrepresented in randomized clinical trials. The use of standardized outcome measures (PASI and DLQI) at predefined timepoints allowed for a comparison of the results with clinical trials and other real-world experiences, while the addition of drug survival analysis provided valuable insights into treatment persistence under routine clinical conditions. Moreover, the systematic collection of previous biologic exposures and treatment durations offered a detailed overview of therapeutic trajectories in real-life psoriasis management.

However, several limitations must be acknowledged. The retrospective nature of this study may introduce information and selection bias, and the relatively small sample size limits the statistical power of subgroup and regression analyses. Missing data at some timepoints may have influenced the precision of longitudinal comparisons. The short observation period (16 weeks), although reflecting the primary assessment window, does not allow for a comprehensive evaluation of long-term effectiveness; the 12-month Kaplan–Meier analysis should therefore be interpreted as an exploratory post hoc assessment rather than a predefined endpoint. Moreover, given the small number of patients with fewer than four prior biologic failures, formal subgroup comparisons were not statistically meaningful and were therefore limited to descriptive observations. Additionally, the absence of a control group precludes direct comparisons with other biologics.

Despite these limitations, this study provides valuable real-world evidence supporting the rapid onset of action, efficacy, and favorable safety profile of bimekizumab, even in patients with long-standing, multi-refractory psoriasis.

## 6. Conclusions

To sum up, our results suggest that bimekizumab may be an effective and well-tolerated therapeutic option for patients with moderate-to-severe psoriasis who have failed multiple previous biologics. Bimekizumab demonstrated rapid onset of action, high rates of complete skin clearance, significant improvement in quality of life, and sustained long-term persistence with a favorable safety profile.

These findings further support the role of bimekizumab as a valuable therapeutic option in the management of multi-failure patients, contributing to a more individualized and sustained disease control in real-life practice. Given the limited sample size, these findings should be interpreted descriptively and warrant confirmation in larger real-world cohorts.

## Figures and Tables

**Table 1:** Baseline characteristics and treatment outcomes of multi-failure patients with moderate-to-severe plaque psoriasis treated with bimekizumab.

Number of Patients			33	
**Age (*years, mean ± SD*)**			56.2 ± 12.8	
**Psoriasis duration (*m*** ***ean ± SD*** **)**			29.1 ± 13.0	
**BMI (*mean ± SD*)**			29.8 ± 5.0	
**Male (*n*, %)**			18 (54.5%)	
**Psoriatic arthritis (*n*, %)**			15 (51.5%)	
**Difficult-to-treat area involvement (*n*, %)**				
Nail			11 (33.3%)	
Scalp			23 (69.7%)	
Genitalia			13 (39.4%)	
**Comorbidities (*n*, %)**				
Metabolic (DM, obesity, dysmetabolic disorders)			13 (39.4%)	
Cardiovascular			11 (33.3%)	
Oncologic			6 (18.2%)	
Hepatic (HBV/HCV)			4 (12.1%)	
Autoimmune/inflammatory			4 (12.1%)	
Endocrine			3 (9.1%)	
Psychiatric			2 (6.1%)	
Other			6 (18.2%)	
**Previously used biologic agents (*n*, %)**				
**Anti-TNF**				
Adalimumab			28 (84.8%)	
Certolizumab			4 (12.1%)	
Etanercept			10 (30.3%)	
Infliximab			11 (33.3%)	
Golimumab			1 (3.0%)	
**Anti-IL17**				
Brodalumab			9 (27.3%)	
Ixekizumab			16 (48.5%)	
Secukinumab			19 (57.6%)	
**Anti-IL12/23**				
Ustekinumab			13 (39.4%)	
**Anti-IL23**				
Guselkumab			13 (39.4%)	
Risankizumab			10 (30.3%)	
Tildrakizumab			8 (24.2%)	
**Treatment duration with prior biologic agents (*months, mean ± SD*)**				
**Anti-TNF**				
Adalimumab			22.1 ± 19.9	
Certolizumab			10.8 ± 8.5	
Etanercept			25.3 ± 18.1	
Infliximab			22.0 ± 20.1	
Golimumab			8	
**Anti-IL17**				
Brodalumab			7.1 ± 3.9	
Ixekizumab			20.4 ± 14.9	
Secukinumab			20.2 ± 18.5	
**Anti-IL12/23**				
Ustekinumab			27.6 ± 33.3	
**Anti-IL23**				
Guselkumab			22.1 ± 19.4	
Risankizumab			15.7 ± 9.1	
Tildrakizumab			11.3 ± 6.7	
**Number of prior biologic therapies (*n*, %)**				
2			1 (3.0%)	
3			1 (3.0%)	
4			14 (42.4%)	
5			15 (45.5%)	
6			2 (6.1%)	
**Efficacy outcomes**				
	**Baseline**	**Week 4**		**Week 16**
**PASI (*mean ± SD*)**	14.5 ± 7.1	2.5 ± 2.9		1.5 ± 4.0
**PASI90 response**	NA	14 (42.4%)		19 (57.6%)
**PASI100 response**	NA	7 (21.2%)		14 (42.4%)
**Percentage of PASI reduction**	NA	−79.7%		−90.4%
**DLQI (*mean ± SD*)**	15.7 ± 5.0	2.3 ± 3.0		2.0 ± 3.6
**Percentage of DLQI reduction**	NA	−86.9%		−84.2%

Data are expressed as mean ± standard deviation (SD) or number (percentage), unless otherwise specified. PASI: Psoriasis Area and Severity Index; DLQI: Dermatology Life Quality Index; PsA: psoriatic arthritis; BMI: body mass index; TNF: tumour necrosis factor; IL: interleukin; NA: not applicable.

## Data Availability

The original contributions presented in this study are included in this article. Further inquiries can be directed to the corresponding authors.
